# Hopfield and Hinton’s neural network revolution and the future of AI

**DOI:** 10.1016/j.patter.2024.101094

**Published:** 2024-11-08

**Authors:** James Z. Wang, Brad Wyble

**Affiliations:** 1 Data Sciences and Artificial Intelligence Section, and Human-Computer Interaction Section, College of Information Sciences and Technology, The Pennsylvania State University, University Park, PA, USA; 2 Department of Psychology, The Pennsylvania State University, University Park, PA, USA

## Abstract

In this opinion piece, the authors, from the fields of artificial intelligence (AI) and psychology, reflect on how the foundational discoveries of Nobel laureates Hopfield and Hinton have influenced their research. They also discuss emerging directions in AI and the challenges that lie ahead for neural networks and machine learning.

## Main text

### Introduction

The 2024 Nobel Prize in Physics was awarded to John H. Hopfield and Geoffrey E. Hinton for their “foundational discoveries and inventions that enable machine learning with artificial neural networks” (ANNs). Their contributions have profoundly shaped modern AI, laying the foundations for technologies that are now embedded in everyday life. Historically, the Nobel Prize has rarely recognized the field of computer science or AI, making this recognition not only a testament to their work but also a milestone for the entire computing community. Their work is also transforming many fields. Notably, the 2024 Nobel Prize in Chemistry recognized the creators of AlphaFold2, a deep learning system that has revolutionized protein structure prediction.

We are grateful for the opportunity to reflect on how Hopfield and Hinton’s work has profoundly influenced our research. Inspired by their contributions, our discussion of emerging opportunities and unresolved obstacles in AI, grounded in our work, aims to further AI’s potential to help address some of society’s most pressing challenges.

### Foundational contributions by Hopfield and Hinton

Both Hopfield and Hinton have had extraordinarily productive careers, making foundational contributions across multiple fields. Hopfield has significantly impacted AI, system biology, and physics, while Hinton’s work spans AI, psychology, and cognitive science.

Hopfield is best known for inventing the Hopfield network, an energy-based associative memory model that converges to stable states through energy minimization.^1^ This was an early attempt to model cognitive processes like memory and pattern recognition and laid the groundwork for future developments in ANNs, influencing modern networks like long short-term memory (LSTM) networks and gated recurrent units (GRUs).

Hinton’s key contributions include co-inventing Boltzmann machines, which were an important step in unsupervised learning and generative models,^2^ and advancing the backpropagation algorithm, which enables the training of deep ANNs.^3^ His lab’s work on AlexNet demonstrated the power of deep learning for large-scale vision tasks.^4^

The development of deep learning can also be attributed to contributions from many other scientists. For instance, Warren McCulloch and Walter Pitts developed the first mathematical model of a neuron. Frank Rosenblatt invented the Perceptron, one of the first models capable of learning from data. Some of the earliest attempts to build a multi-layer neural network were made by Alexey G. Ivakhnenko and Valentin G. Lapa. The term deep learning was introduced to the AI community by Rina Dechter.

### Personal reflections

#### James Z. Wang

In my 30-year AI research career, Hopfield and Hinton’s work has been a consistent source of inspiration. Although I began in mathematics, I quickly found my passion in using mathematics and computing to explore new possibilities. With guidance from Gio Wiederhold, I transitioned to the medical AI PhD program created by Ted Shortliffe.

In the 1990s, AI was considered a struggling field, often referred to as an “AI Winter,” because classical approaches, including early ANNs, couldn’t scale to real-world problems. ANNs were taught as historical topics, while methods like Markov processes, classification trees, and support vector machines took center stage. In 2002, we published Automatic Linguistic Indexing of Pictures (ALIP), formulating image semantic annotation as a statistical classification challenge. We used a multiscale hierarchical hidden Markov model to demonstrate that computers could learn to annotate images with linguistic terms selected from a pre-defined “dictionary.”^5^ Few believed such tasks were achievable at the time. After optimizing the algorithm for real-time performance in 2006,^6^ I shifted focus, not anticipating the breakthroughs that would follow.

Hinton’s lab’s 2012 achievement in image classification^4^ was striking. It defied the prevailing belief that ANNs couldn’t handle large, complex problems. Their use of GPUs to scale ANNs through vectorization and parallelism reminded me of CRAY supercomputers. I was astonished by the model’s accuracy, knowing firsthand the challenges of image classification. Convolutional neural networks, with their ability to automatically extract hierarchical features, were pivotal.

**Figure 1:**
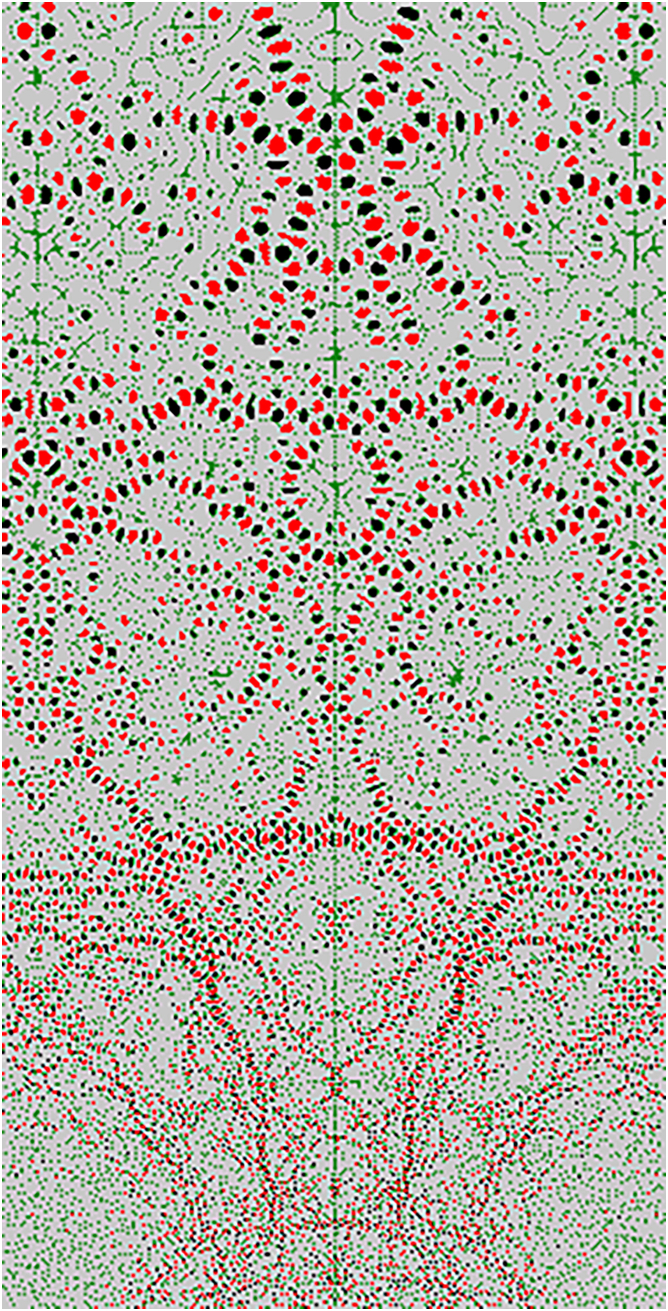
A visualization of the quantum mechanical behavior in the Lobachevsky space Image generated with a CRAY supercomputer in the early 1990s. The research was led by Dennis Hejhal (University of Minnesota).^8^

Over the past two decades, I’ve focused on modeling visual aesthetics, emotion, and artistic style—areas that remain relatively underexplored.^7^ This interest originated from my undergraduate research with Dennis Hejhal, where I used CRAY supercomputers to explore Lobachevsky space (Figure 1),^8^ sparking my fascination with the intersection of mathematics, computing, and art. While I expected machine learning to gradually improve logical problem-solving, I recognized that aesthetics, emotional intelligence, and creativity remain largely uncharted frontiers in AI.

The resurgence of ANNs prompted me to revisit certain ideas and adopt new methodologies. Collaborating with colleagues, we’ve made significant progress, utilizing tools like Transformers, CLIP, and diffusion models. In addition to core research areas, we’ve developed ANN-based methods for analyzing placenta images to improve maternal and neonatal health, diagnosing strokes from patient videos, and segmenting 3D images to study drought-resistant crops.

Hinton’s breakthroughs continue to shape my outlook. Despite significant advancements, AI systems are still far from their full potential. My collaborators and I are tackling fundamental challenges like improving explainability in medicine and reducing the reliance on vast amounts of training data. In the following reflection, Wyble will elaborate on the latter.

#### Brad Wyble

In high school, I purchased *Explorations in Parallel Distributed Processing*, co-authored in part by Hinton. The idea that computers could *learn* using simulated neurons was transformative for me and set the stage for my interest in AI. However, I entered college in the early 1990s, a low point for interest in trained neural networks, and therefore, I studied biologically inspired neural networks. My models were hand-tuned to reflect neurobiological mechanisms and attractor dynamics, as theorized in Hopfield networks.

Later, the field of neural networks re-emerged as a method to understand visual processing as I discovered in the early work of Thomas Serre, who built models of visual processing that simulated the location-invariance of neurons within the monkey visual system. I was captivated by the ability of such models to classify natural images, years before Hinton lab’s work on AlexNet was published.^4^ These models provided me with a functional intuition for how neurons in my own brain allowed me to recognize objects, a process that I previously considered cognitively impenetrable.

Hinton and other AI pioneers provided a way to automate the training of such networks at scale, which made them computationally accessible. Consequently, the applied and theoretical aspects of deep learning bloomed in the years that followed, and I used deep learning to better understand how the mind represents visual information in a distributed manner. Using backpropagation to train variational autoencoders simulates how perceptual systems like the human ventral stream might learn to process visual stimuli without supervised training. Our lab built a new theory of human working memory that used the layers of such autoencoders as the computational substrate for mental representations of visual forms,^9^ improving on previous models of working memory that simulated only individual features such as color and orientation. The automated and highly efficient backpropagation algorithms enabled this research by allowing us to explore the space of models efficiently.

**Figure 2:**
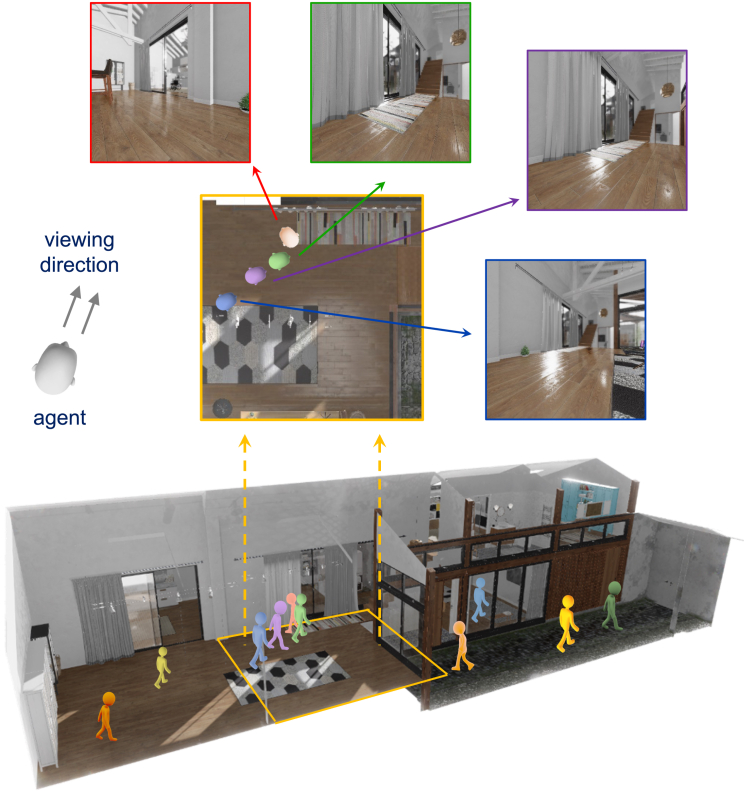
An illustration of the core idea of our spatial contrastive learning approach, where an agent navigates a 3D virtual environment The bottom shows the agent’s trajectory and viewing direction, while the top images represent different viewpoints captured at various positions. These simulated perspectives provide the spatial context necessary for training the model in visual pattern recognition.^10^

As impressive as deep learning is, there still remain stark challenges—notably the reliance on enormously large training sets to approximate human performance, even in core vision tasks like classification. The appetite of these models for data, particularly those trained with unsupervised contrastive learning, stands in contrast to the ability of human children to learn the visual statistics of the real world with a fraction of the total amount of visual input. Recently, I have been working with the James Wang lab to improve the efficiency of training such models by incorporating environmental context. This idea is inspired by the observation that humans perceive the world with a sense of space around them whereas deep learning models are trained without context. Their training sets are randomly shuffled to remove spurious correlations between image sequences, but this shuffling eliminates the possibility of exploiting the deeper structure that exists from explorations of the natural world. We developed methods that train better models using structured datasets acquired from exploration of a virtual environment (Figure 2).^10^

### Emerging directions and unsolved challenges in AI

While valid concerns around safety and unintended consequences have led many to advocate pausing giant AI experiments, advancing AI to tackle urgent global challenges—such as climate change, public health crises, poverty, food security, environmental degradation, mental health, population growth, and education—is crucial, provided innovation aligns with humanity’s core values. We see value in remaining cautiously optimistic about our ability to collaborate effectively with AI.

ANNs excel at identifying patterns in vast datasets, which explains their effectiveness in language processing and computer vision tasks involving structured, low-entropy environments. However, the world is filled with subjectivity, ambiguity, and individual differences. AI’s ability to move beyond task-specific learning to handle such complexity will define its future progress.

Hopfield and Hinton demonstrated that breakthroughs often arise from the convergence of different scientific fields. We see opportunities for AI to integrate with other disciplines, which may help overcome some of its limitations. For instance, climate modeling demands staggering amounts of data—from sensors, satellites, and human activities—across dynamic temporal scales. AI’s ability to handle such interconnected systems will affect our ability to predict and mitigate climate risks. Similarly, AI holds transformative potential in healthcare, but models—often trained on healthy populations—must adapt to the personalized nature of diagnoses and treatments to be truly effective.

In biology, AI faces challenges in generalization. Biodiversity encompasses a vast array of species, genetic diversity, and ecosystems, yet models trained on one part of this landscape often fail elsewhere. Likewise, AI applications in psychology are complex due to factors like personality, culture, emotion, and context. Modeling this richness requires high-quality data, but the subjective nature of many aspects, like emotion, complicates data collection and interpretation.^7^ Similar issues exist in the arts, where AI’s exploration of stylistic patterns inspires innovation. Art is inherently creative and subjective, with many unsolved art historical questions that no single algorithm can address.^11^^,^^12^ Moreover, art-related research must be conducted ethically to avoid exploiting human artists’ work.

Beyond practical applications, AI invites deeper intellectual exploration. Galileo described mathematics as the language in which the universe is written. To genuinely test AI’s intelligence, we might wonder whether it’s even possible to train AI to understand the universe beyond human knowledge, generate original abstract conjectures, or assist in proving long-standing problems like the Riemann hypothesis or P vs. NP. As AI surpasses humans in some areas, philosophical reflections become increasingly important. Advancements may catalyze a societal shift toward deeper reflection on human purpose, potentially redefining success to emphasize spiritual development and connections with oneself and others.

Despite progress, many challenges persist in AI research. The success of technologies like ChatGPT and autonomous vehicles has ignited widespread enthusiasm. Scholars, including Hopfield and Hinton, along with business leaders, have suggested that AI could surpass human intelligence, potentially delivering significant benefits or catastrophic outcomes.^13^ However, we believe AI is unlikely to surpass human intelligence soon, and it may never do so. AI’s key limitations—lack of explainability, bias, hallucination, difficulty handling out-of-distribution data, reliance on massive datasets, catastrophic forgetting, vulnerability to attacks, high computational costs, and inadequate symbolic reasoning—are well documented. Addressing these is essential for responsible AI development, yet even with improvements, fundamental constraints remain.

AI relies on learning from statistical patterns, symbolic processing, and optimization, whereas human intelligence is *multidimensional*, drawing on imagination, judgment, emotional intelligence (EQ), and consciousness.

A significant gap lies in understanding narratives. AI can list objects in a scene or describe actions in a video but struggles to grasp deeper meaning or themes like betrayal, generosity, sacrifice, or seduction. Humans naturally create and comprehend complex narrative structures, with thousands of tale-types and allomotifs documented in writing, film, and folktales within recent history by folklorists like Hans-Jörg Uther.

EQ is another area where AI falls short. Humans have evolved to manage emotions—both their own and others’—and studies show EQ often plays a larger role than IQ in success.^14^ While AI has made progress in emotion recognition,^7^ it lacks lived experience and cannot truly experience emotions or apply them in decision-making. Embodied learning, where agents interact physically with their environment, offers some promise but requires breakthroughs across multiple disciplines to capture and replicate the depth of human EQ.

**Figure 3:**
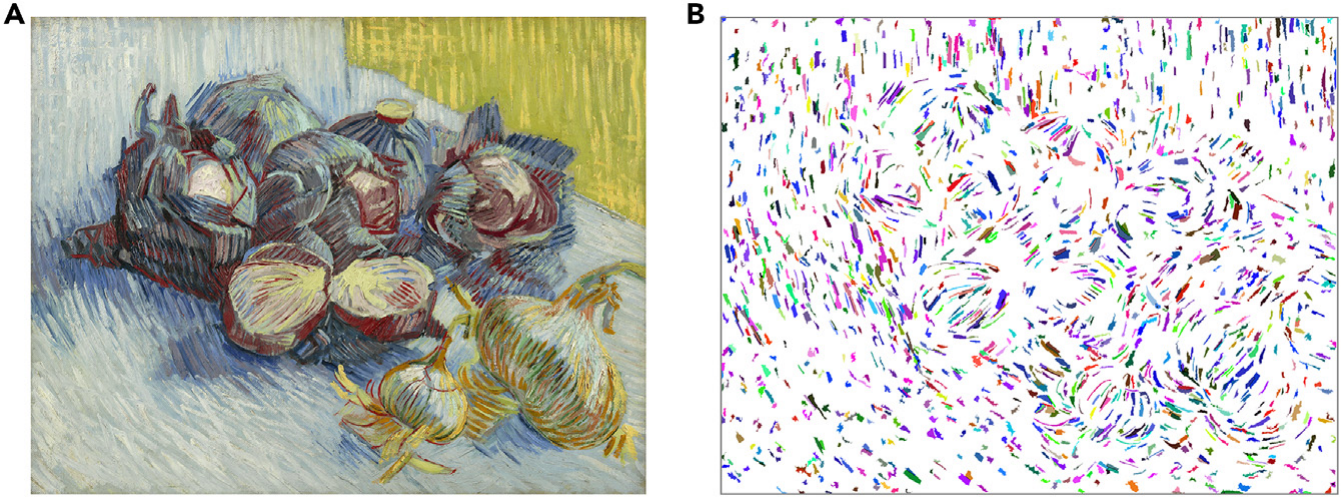
Automatic brushstroke extraction reveals rhythmic structures in Vincent van Gogh’s paintings (A) Red Cabbages and Onions, Paris, 1887, oil on canvas. Image courtesy of the Van Gogh Museum, Amsterdam, the Netherlands (Vincent van Gogh Foundation). (B) Brushstrokes are extracted from this painting using a computer algorithm, with random colors assigned individually to each brushstroke. Image provided by the James Z. Wang Research Group.^12^

Creativity presents additional challenges for AI. Humans generate novel ideas, as evident in mathematics and art. Throughout history, certain individuals, often regarded as geniuses, have developed unique styles or revolutionary ideas independently. For example, Vincent van Gogh’s work, which was created over just a decade, continues to reveal new insights (Figure 3).^12^ Similarly, John Constable’s cloud studies reflect early meteorological understanding.^11^ AI lacks the imagination and cross-disciplinary creativity necessary for such innovation.

Human intuition and common sense, which enable quick decision-making without explicit rules, remain elusive for AI. Mathematicians may spend hundreds of pages of logical deductions to prove a conjecture—a task that would be combinatorially infeasible if all possibilities had to be exhaustively tried. Neural AI systems lack abstract thinking and symbolic reasoning capabilities.

In ethical and moral reasoning, humans incorporate complex considerations into everyday decisions, often instantaneously. AI, constrained by predefined rules or learning corpora, struggles with unprecedented or morally complex situations, raising safety concerns about responsible behavior in unpredictable scenarios. Another limitation is AI’s lack of intrinsic motivation and self-determination. Humans can devote decades to a goal, driven by purpose and experience, as exemplified by mathematician Yitang Zhang, who worked for many years on number theory problems without recognition.

Human intelligence is broad and adaptable, allowing us to integrate various capabilities to tackle complex tasks and make decisions even in unfamiliar domains. These competencies have empowered us to create great works of art, develop astounding technologies, and explore the moon and ocean floor. The ultimate question remains, “can AI truly capture the complexity of human creativity and emotional depth?”

### Conclusion

The groundbreaking work of Hopfield and Hinton has revolutionized AI and will inspire generations. While AI has achieved significant progress, challenges remain. The future of AI will depend on its thoughtful integration into solving global issues and enriching human life, ensuring that innovation remains aligned with human values and advances the well-being of society.

## Acknowledgments

The authors thank their collaborators, advisees, and past mentors for valuable discussions and support. J.Z.W.’s research is supported primarily by the NSF under grant nos. 2015943, 2205004, 2216127, 2234195, and 2327730; the NIH under grant no. R01EB03130; the National Endowment for the Humanities under grant no. HAA-287938-22; the Amazon Research Awards Program; and the Burroughs Wellcome Fund. B.W.’s research is supported primarily by the NSF under grant no. 2216127. The views and conclusions expressed are those of the authors and do not necessarily reflect the views of the funding agencies.

## Declaration of interests

J.Z.W. is named as an inventor on patents and patent applications related to the technologies discussed in this article. These patents are held by The Penn State Research Foundation. The opinions expressed here have not been influenced by these inventions.

## Declaration of generative AI and AI-assisted technologies in the writing process

During the preparation of this work, the authors used ChatGPT-4o in order to improve the readability of the manuscript. After using this tool, the authors reviewed and edited the content as necessary and take full responsibility for the content of the published article.
